# A Review of Historical Changes of Tropical and Extra-Tropical Cyclones: A Comparative Analysis of the United States, Europe, and Asia

**DOI:** 10.3390/ijerph19084499

**Published:** 2022-04-08

**Authors:** Yui-Yip Lau, Tsz-Leung Yip, Maxim A. Dulebenets, Yuk-Ming Tang, Tomoya Kawasaki

**Affiliations:** 1 Division of Business and Hospitality Management, College of Professional and Continuing Education, The Hong Kong Polytechnic University, Hong Kong, China; yuiyip.lau@cpce-polyu.edu.hk; 2 Department of Logistics and Maritime Studies, The Hong Kong Polytechnic University, Hong Kong, China; t.l.yip@polyu.edu.hk; 3 Department of Civil & Environmental Engineering, College of Engineering, Florida A&M University-Florida State University (FAMU-FSU), Tallahassee, FL 32310-6046, USA; mdulebenets@eng.famu.fsu.edu; 4 Department of Industrial and Systems Engineering, The Hong Kong Polytechnic University, Hung Hom, Hong Kong, China; 5 Department of Systems Innovation, School of Engineering, The University of Tokyo, Tokyo 113-8656, Japan; kawasaki@sys.t.u-tokyo.ac.jp

**Keywords:** climate change, tropical cyclones, adaptation, resilience, humanitarian logistics

## Abstract

Tropical cyclones are highly destructive weather systems, especially in coastal areas. Tropical cyclones with maximum sustained winds exceeding 74 mph (≈119 kph) are classified as typhoons in the Northwest Pacific, whilst the term ‘hurricanes’ applies to other regions. This study aims to investigate the general characteristics of the most devastating and catastrophic tropical cyclones in the USA Europe, and Asia. To achieve the study objectives, the three most devastating typical tropical cyclones in each region were selected. The tropical cyclones were examined based on various features, such as the number of deaths, minimum pressure, highest wind speed, total financial losses, and frequency per year. In contrast to Europe and Asia, the USA has recorded the highest number of catastrophic tropical cyclones. The damage induced by hurricanes Katrina, Harvey, and Maria in the USA totalled approximately USD USD 380 billion. In addition, the present research highlights the demand to improve the public attitude and behaviour toward the impact of climate change along with the enhancement of climate change alleviation strategies. The number of intense tropical cyclones is expected to rise, and the tropical cyclone-related precipitation rate is expected to increase in warmer-climate areas. Stakeholders and industrial practitioners may use the research findings to design resilience and adaptation plans in the face of tropical cyclones, allowing them to assess the effects of climate change on tropical cyclone incidents from an academic humanitarian logistics viewpoint in the forthcoming years.

## 1. Introduction

Climate refers to long-term average weather conditions. Regardless of whether the climate is stable or dynamic, it affects all living organisms on the planet [1]. A stable climate provides people, plants, and animals with reliable living conditions. According to [1,2,3,4], the climate affects our daily lives. In other words, climate change will force people’s daily lives to also change. Some of the notable examples at present are the altering socio–ecological landscapes, green economy/low carbon phenomenon, agricultural diversification and crops patterns, demand for energy consumption and heating buildings, shipping activities, and infrastructure maintenance. Climate change is a good precedent for research in several disciplines as it entails a ‘lasting process’, even constituting a catastrophic risk to human welfare [5]. According to Birkmann et al. [6], ‘climate change impacts include multi-hazard phenomena, such as the simultaneous occurrence of sudden-onset hazards and creeping changes’. The changes in weather patterns directly influence the Earth’s flora, consequently affecting all living organisms, including animals and humans [1].

Tropical cyclones are identified as catastrophic meteorology systems, especially in vulnerable shore-side regions [7]. Hurricanes and typhoons are viewed as tropical cyclones under similar weather circumstances. Tropical depressions are commonly described as the lowest types of tropical cyclones. At the growth stage, the tropical cyclone shifts to a tropical storm when the maximum sustained wind reaches 39 mph [8]. At the maturity stage, assuming the maximum sustained wind further increases to 74 mph, the tropical cyclone is categorised either as a hurricane or a typhoon depending on the origin; in particular, the term ‘typhoon’ is common in the Northwest Pacific, whereas the term ‘hurricane’ applies to other regions [8]. Tropical cyclones have higher recurrence rates compared with other hazards, such as earthquake disasters, and the accumulated losses resulting from tropical cyclone disasters are considerable. As such, tropical cyclone disasters are the most severe types of natural disasters and have emerged as a notable issue. Numerous tropical cyclones produce a wave of severe weather incidents, including widespread flooding and intense storms and rainfall. These weather conditions bring unfavourable effects, namely, life threats, health hazards, critical infrastructure damage (i.e., damage to ports, cargo terminals, and highways), journey interruption, huge financial losses, fatalities, serious destruction to buildings, impact on social wellbeing, deviation of routing and navigation, cargo damage, and cargo casualties [9,10,11]. Consequently, humanitarian logistics has been inclined toward assisting people in their survival during and after a disaster. However, damaged transportation infrastructures and communication facilities usually restrict humanitarian support access to regions affected by hurricanes and super typhoons [12]. 

Over the past few decades, the continuing climate change has modified the categorisation of tropical cyclones in terms of their movement frequency, strength, and prevailing conditions [13,14,15,16]. According to the *Intergovernmental Panel on Climate Change AR6 (Sixth Assessment Report) Report* [17], large-scale atmospheric circulations (e.g., monsoon or Walker and Hadley circulation) remarkably influence tropical cyclones. Furthermore, changes in sea surface temperature and warming patterns induce regional tropical cyclone activities, making them highly unpredictable. As further emphasised by the report, (1) the frequency of rapid intensification incidents and the portion of main tropical cyclone intensities have both expanded worldwide in the last four decades, (2) the common locations where tropical cyclones reach their peak wind intensity have shifted poleward in the western North Pacific Ocean since the 1940s and (3) the tropical cyclone translation speed has moderated over the USA since the 1900s. A slow translation speed will pose a threat to inland and coastal areas. 

The relationships between tropical cyclone activities, climate change, and potential impact on human activities have become a ‘hot’ agenda in the International Workshop on Tropical Cyclones and the Paris Agreement [18]. Subsequently, [18,19,20,21] examined whether climate change affects tropical cyclones. However, the aforementioned studies only focused on pure conceptual or theoretical matters, suggesting that their respective findings may not produce an unbiased vortex with physical reliability [7]. Other researchers, such as [22,23,24] focused on using sophisticated numerical weather models to forecast weather impacts. Shu-Dong, Juan-Juan and Bin [7] criticised that a lack of knowledge of the typhoon system and thermodynamic mechanisms lead to an inaccurate explanation of the movement and structure of a typhoon. In addition, the majority of the research on tropical cyclones is mainly focused on a simple single case study, e.g., Macau [14], New York City [25], Taiwan [10], and Shanghai City [26]. Besides the aforementioned works, [1,5] addressed the stakeholder perceptions on the effectiveness of climate adaptation actions. 

Several catastrophic tropical cyclone events have led to physical and social vulnerability. Buildings, infrastructure, and food crops are often substantially damaged along with the falling of numerous trees [27]. Suffering and deaths are also induced by objects that block the rescue of injured persons [28]. Water supplies become contaminated with saltwater or are even destroyed. Telecommunications or mobile phone networks covering vast areas stop functioning. Furthermore, almost all wastewater is unmanaged in hazard-prone areas. People living in low-lying areas are vulnerable because of flooding, either from heavy rain or sea and landslides. Compared with individuals living in urban areas, people in remote/rural locations are at a higher risk because they are less accessible and more disconnected, and they have poor infrastructure, fragile livelihoods, weak early warning capabilities, insufficient cyclone shelters; more alarming, they suffer from long distances to cyclone shelters and have slow access to external aid and rescue from humanitarian services [29,30,31]. A detailed analysis of the typhoon impacts is often overlooked in many studies. Refs. [32,33] have performed an analysis of the volatility of short-term changes in seasonal typhoon activity and found that these aspects may not represent a holistic understanding of the current situation. 

Acknowledging the aforementioned drawbacks in the state-of-the-art and existing research, this study investigates the key characteristics and trends of tropical cyclones in the USA Europe, and Asia since 2000. By examining the meteorological contexts, the structure of the typhoons and hurricanes, and the major attributes of typhoons and hurricanes (i.e., highest wind speed, year of occurrence, lowest pressure, total monetary losses, total deaths, and total rainfall), we highlight the main meteorological factors that have caused disasters and their consequences. We believe that this study will be useful to researchers, industrial practitioners and the human society overall, as the outcomes of this work can assist with addressing the adverse impacts of climate change on tropical cyclone circumstances, along with designing resilience and adaptation strategies in response to tropical cyclones in the coming years. 

The manuscript is divided into five main sections. In Section 1, the study provides research settings, context, and objectives. In Section 2, the study adopts the concept of humanitarian logistics in typhoon events. Section 3 elaborates on the data sources employed in this study. Based on the adopted data sources, we illustrate the representative case studies of major tropical cyclones for the USA Europe, and Asia in Section 4. A detailed discussion of the findings revealed during the case study analysis is presented in Section 5. Section 6 provides the main conclusions of this study and outlines some limitations that can be addressed as part of future research.

## 2. Humanitarian Logistics: Typhoon Events

Understanding the typhoon events from the logistical perspective will provide valuable knowledge for hazard management. Typhoon events can be formulated as a logistics problem, and they have several logistics attributes (Table 1).

**Figure 1:**
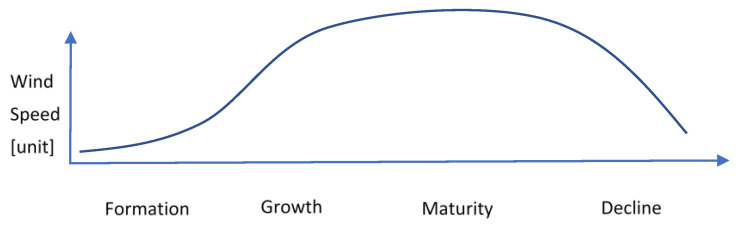
Product life cycle (PLC) of a typhoon event.

A typhoon event can be divided into four main stages of the product life cycle, namely, formation, growth, maturity, and decline (Figure 1). Figure 1 illustrates the concept of the product life cycle. The proposed life cycle concept can be directly used for typhoon analysis, particularly, in generating valuable lead time for significant humanitarian logistics strategies after the typhoon emerges. As such, Figure 1 may help to explain the interface between typhoon events and humanitarian logistics. At the formation stage, a generic plan of humanitarian logistics of high flexibility is needed. During the growth stage, plans become more specific, as objectives of humanitarian logistics become more definite and resources become more committed. Humanitarian logistics is the combination of humanitarianism and logistics which is defined as ‘the process of planning, implementation, and control efficiently, focusing on low-cost flow and storage of goods and materials and related information, from point of origin to point of consumption to relieve the suffering of vulnerable people’. Its role encompasses a range of activities, including the planning, preparation, transportation and acquisition, storage, monitoring, and tracking [34]. As such, humanitarian logistics aims at keeping good living conditions and the health of affected populations [35]. When a typhoon is mature, predictability is specific plans of humanitarian logistics are appropriate. Resource availability is certain. From maturity to decline, the available resources should be moved to the locations needed. Indeed, some studies have indicated the necessity of investigating the specific characteristics of post-typhoon humanitarian logistics to develop a proper response [12].

**Figure 2:**
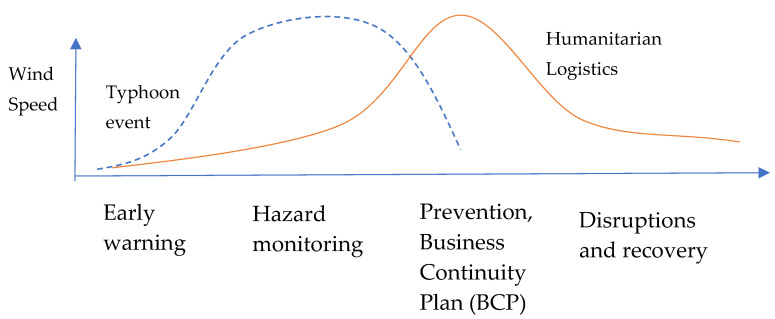
The interface of a typhoon event and humanitarian logistics.

In general, typhoons are meteorological phenomena that can cause natural disasters. When a typhoon happens, humanitarian logistics operations are proposed to give quick assistance to victims, deliver supplies, remove dead bodies, give medical assistance and shelters, and save the disabled. As such, delays in giving help and distributing supplies may potentially cause the loss of lives [36]. Lau et al. [37] indicated that in case of lengthy typhoon events, sufficient funds and resources are vital. Humanitarian logistics is a critical element of the effectiveness of humanitarian action. This is because it fosters the flow of services and goods in a supply chain. To the best knowledge of the authors, climate change induces a high frequency, large scale, and strong power of typhoons from year to year [1,5,38]. The number of victims per year will rise due to the prolonging of tropical cyclone life span, expanding the distance travelled after landfall and intensifying storm destructiveness over land [39]. Hence, particular actions should be designed, planned and implemented, in which humanitarian logistics operations are flexible, reliable, and agile during the time of a particular rescue. In other words, humanitarian logistics should more emphasise pre-disaster preparedness instead of post-disaster support [40]. As expected, the number of victims and economic losses can be significantly reduced [36]. Nevertheless, humanitarian logistics experiences particular difficulties and challenges depending on the location, type, and strength of typhoons in different typhoon events [35]. The interface between a typhoon event and humanitarian logistics is provided in Table 2 and Figure 2.

## 3. Data Sources

In this study, we collected published information, archives, and historical data from newspapers (e.g., BCC News), magazines (National Geographic), scientific articles from academic journals and tropical cyclone reports from professional bodies and governments (e.g., National Hurricane Center, National Aeronautics and Space Administration, Hong Kong Observatory, Macau Geophysical and Meteorological Bureau, to name a few). All the adopted sources provide reliable and rich information that is open to the public. As such, the quantitative data can foster the researchers to pursue an in-depth exploration of the tropical cyclone events that previously happened in the USA Europe, and Asia. In addition, the quantitative data can help the researchers to discover the reasons behind tropical cyclone occurrence and the consequences of tropical cyclones. Based on the research conducted by [41], this study selected tropical cyclone events by considering the purposeful sampling strategies. The selected cases can provide rich information, generalise meaningful study results, exhibit uncommon demonstrations of the experience and serve as examples of extreme events. The selection of tropical cyclone events was also conducted following the guidelines provided by [42], and they have been directly regarded as the main research objectives and research questions of the present study.

## 4. Results

### 4.1. Hurricanes in the USA 

**Figure 3:**
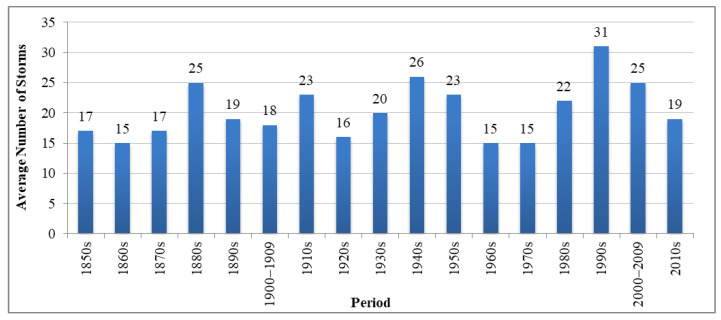
Number of recorded storms in the USA since the 1850s.

**Figure 4:**
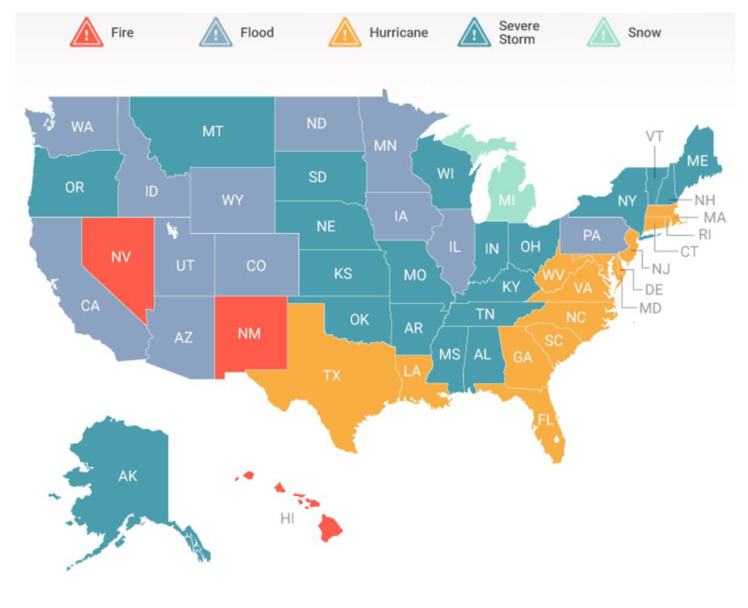
Natural disaster declarations in the USA states. Source: FEMA [52].

Tropical cyclones occur quite frequently in many states of the USA. Figure 3 presents the annual average number of storms over the decade that affected the USA since the 1850s, in which more than 15 storms on average were recorded every year [43]. The highest average of 31 storms was recorded in the 1990s. According to Figure 4, most hurricanes occur on the USA East Coast. As for the USA West Coast, hurricanes are generally not observed; however, other types of natural hazards happen quite frequently (e.g., fire, flooding, and severe storms). Florida, Texas, North Carolina, Georgia, Louisiana, and South Carolina are considered the states that have been affected the most due to hurricanes. The list of costliest hurricanes in the USA since 2000 is presented in Table 3. Along with the monetary losses, Table 3 also includes the information regarding the year of occurrence, total deaths, lowest pressure, highest wind speed, and total rainfall. The lowest pressure and the highest wind speed for the landfall are also shown in the table. Table 3 was prepared using the data reported in [43,44,45,46,47,48,49,50,51]. If different monetary losses due to hurricanes have been reported by the considered sources, then the maximum values of the monetary losses can be adopted. 

The collected data indicate that Katrina, Harvey, and Maria are the costliest hurricanes that occurred after 2000 in the USA. The damage caused by these hurricanes totalled approximately USD USD 380 billion. Hurricane Maria is considered the deadliest one, as it resulted in 3059 fatalities. However, while Hurricane Wilma was not as deadly as the other hurricanes (a total of 52 fatalities; Table 3), it resulted in more than USD USD 24 billion worth of damage. Hurricanes Wilma and Irma had the highest speed of sustained winds exceeding 180 mph. On the other hand, Sandy had lower sustained wind speeds of up to 115 mph. Furthermore, the lowest pressure was recorded for Wilma, Katrina, and Maria. Another important pattern consists in those 5 out of the 10 costliest USA hurricanes since 2000 that occurred in 2017 and 2018 and caused over USD USD 340 billion worth of damage. In particular, Harvey, Maria, and Irma occurred in 2017, whereas Florence and Michael struck the USA coastal areas in 2018 [43,44,45,46,47,48,49,50,51]. The following sections of the manuscript provide more information regarding the top three costliest USA hurricanes, including Katrina, Harvey, and Maria. 

#### 4.1.1. Hurricane Katrina

Hurricane Katrina formed on 23 August 2005 as a tropical depression over the south-eastern Bahamas. The depression intensified the following day and became a tropical storm [43]. When Katrina reached the Gulf of Mexico on 26 August, it rapidly intensified into a category 5 hurricane. On 28 August, Katrina approached the Gulf Coast with a minimum central pressure of 902 hPa and a maximum speed of sustained winds of up to 175 mph. Based on the pressure measurement, Katrina is recognised as one of the most intense Atlantic hurricanes in USA history. On August 29, Katrina made its second landfall near Buras-Triumph (Louisiana) with lower sustained winds of up to 125 mph. The hurricane dissipated on 31 August in the Eastern Great Lakes region. The following areas were affected by Katrina: the Bahamas, Florida (USA), Cuba, Louisiana (USA), Mississippi (USA), Alabama (USA), Eastern USA, and Eastern Canada. As indicated earlier, Hurricane Katrina caused 1836 fatalities and USD 160.0 billion worth of damage. Most of the fatalities and property damage were reported in Louisiana. Katrina’s storm surge resulted in breaches of various flood protection structures. Approximately 80% of New Orleans (Louisiana) was flooded. The flooding of New Orleans was primarily caused by the failure of floodwalls. Along with Louisiana, substantial property damages were reported in coastal areas of Mississippi and Alabama [43,44,45,46].

#### 4.1.2. Hurricane Harvey

Hurricane Harvey formed as a westward-moving tropical wave and became a tropical depression on 17 August 2017 in the vicinity of Barbados. The same day, a tropical depression intensified into a tropical storm [43]. The maximum sustained winds of 45 mph were observed in the Caribbean Sea on 18 August. Harvey started a rapid intensification after 23 August and became a category 4 hurricane on 24 August with a minimum central pressure of 937 hPa and a maximum speed of sustained winds of up to 130 mph. On 23 August, Hurricane Harvey made landfall on San Jose Island at full intensity. Three hours later, the hurricane made its second landfall on Rockport (Texas) at a slightly weakened intensity. Harvey started weakening after its landfall and returned to the tropical storm state on 26 August. The last hurricane landfall occurred on 30 August in Cameron (Louisiana), and the maximum speed of sustained winds did not exceed 45 mph. Hurricane Harvey fully dissipated on 2 September. The following areas were affected by Harvey: Windward Islands, Guyana, Suriname, Nicaragua, Belize, Cayman Islands, Honduras, Yucatán Peninsula, Eastern USA, and Southern USA. As indicated earlier, Hurricane Harvey caused 107 fatalities and USD 125.0 billion worth of damage. Loss of electricity, flooding, and infrastructure damage were reported in the Caribbean and Latin America. Texas and Louisiana were impacted the most by Harvey. In particular, the hurricane killed 103 people in Texas, whilst 336,000 people were left without electricity. Approximately 185,000 homes were damaged in Texas, and 9000 homes were destroyed. Heavy rainfall and flooding were reported in Louisiana [43,44,45,46].

#### 4.1.3. Hurricane Maria

Hurricane Maria originated from a tropical wave and developed into a tropical depression on 16 September 2017 in the vicinity of Barbados. Maria started gradually strengthening and became a hurricane on 17 September [43]. On 20 September, Maria reached its peak intensity when entering the vicinity of Puerto Rico with a minimum central pressure of 908 hPa and a maximum speed of sustained winds of up to 175 mph. Hurricane Maria substantially weakened after passing Puerto Rico but was able to intensify again to a major hurricane with sustained winds of up to 125 mph when approaching Hispaniola on 22 September. Significant fluctuations in the intensity levels were observed in the next few days, and the hurricane gradually weakened whilst nearing the USA East Coast. Maria weakened to a tropical storm on 28 September and completely dissipated on 2 October. The following areas were affected by Maria: Lesser Antilles, Dominican Republic, Puerto Rico, Haiti, the Bahamas, Turks, and Caicos Islands, and Southeastern USA. As indicated earlier, Hurricane Maria caused 3059 fatalities and USD 91.6 billion worth of damage. Substantial losses were reported in Puerto Rico. Approximately 80% of agricultural land was lost. Approximately 18 million coffee trees were destroyed. More than 3.4 million were left without electricity in Puerto Rico. Large waves, winds of up to 40 mph, beach erosion, and flooding were reported in North Carolina (USA) [43,44,45,46].

### 4.2. Storms in Europe

Major hurricanes are not common in European countries. In general, major hurricanes do not form east of the 30th meridian west, and those hurricanes that form under this configuration typically continue in the westerly direction. Nevertheless, several extra-tropical cyclones have caused substantial damage in various European countries. European cyclones typically have strong winds and heavy rainfall. In some instances, European cyclones may even have snowfall. Hurricane Vince (2005) is considered the only modern fully tropical storm that directly affected Europe (Madeira Islands, Portugal, and Spain). However, no fatalities and minimal property damage were reported after Hurricane Vince. European cyclones have various paths and affect different countries differently [53,54]. The countries that are located in the Atlantic coastal areas (e.g., Spain, Portugal, France, Germany, Ireland, United Kingdom, and Norway) are generally impacted more than others. The list of costliest storms in Europe since 2000 is presented in Table 4. Along with the monetary losses, Table 4 also includes the information regarding the year of occurrence, maximum wind speed, lowest pressure, total deaths, and total rainfall. The lowest pressure and the highest wind speed are reported concerning the moment of landfall. Table 4 shows the data collated from the literature [55,56,57,58,59,60,61,62,63]. 

The collected data imply that Kyrill, Xynthia, and David are the costliest storms that occurred after 2000 in Europe. The damage caused by these storms totalled approximately USD 10.3 billion. Storm Xynthia is considered the deadliest as it resulted in 63 fatalities. On the other hand, Storm Andrea caused only 1 fatality; however, the total monetary losses associated with the storm comprised approximately USD 350 million. Storms Kyrill and Lorenzo had the highest wind speeds of up to 160 mph. On the other hand, Storm Gudrun had lower wind speeds of up to 103 mph. Furthermore, the lowest pressure was recorded for Lorenzo and Ciara. Another important pattern consists in those 4 out of the 10 costliest European storms since 2000 that occurred between 2018 and 2020 and caused over USD 5.5 billion worth of damage. In particular, Eleanor and David struck Europe in 2018, whereas Lorenzo and Ciara occurred in 2019 and 2020, respectively. The following sections of the manuscript provide more information regarding the top three costliest European storms, including Kyrill, Xynthia, and David [53,54,55,56].

#### 4.2.1. Storm Kyrill

Storm Kyrill formed on 15 January 2007 in the vicinity of Newfoundland (Canada). The storm moved across the entire Atlantic Ocean and reached the coastal areas of Great Britain and Ireland by the evening of 17 January. Kyrill made landfall on the Dutch and German coastal areas on 18 January and then started moving toward Poland and the Baltic Sea with a minimum central pressure of 960 hPa and a maximum wind speed of up to 160 mph [55]. After crossing Poland, Kyrill moved to Northern Russia with a weakened intensity. The storm fully dissipated on 24 January. The following areas were affected by Kyrill: Ireland, United Kingdom, Norway, Netherlands, France, Germany, Belgium, Luxembourg, Poland, Russia, and others. As indicated earlier, Storm Kyrill caused 53 fatalities and USD 4.70 billion worth of damage. Western Europe was affected the most, especially the United Kingdom and Germany. Along with fatalities, power outages, significant public transport disruptions, and damage to private and public buildings were also reported. More than 25,000 homes experienced power outages in Southern England. The coastal areas of Western Europe faced flooding issues. A significant number of fatalities were recorded in Ireland, Netherlands, and Poland.

#### 4.2.2. Storm Xynthia

Storm Xynthia formed on 26 February 2010 and crossed Western Europe between 27 February and 1 March 2010 with a minimum central pressure of 967 hPa and a maximum speed of sustained winds of up to 142 mph. The storm fully dissipated on 7 March [55]. The following areas were affected by Xynthia: Portugal, Spain, France, Belgium, Germany, England, Denmark, Poland, and Sweden. As indicated earlier, Storm Xynthia caused 63 fatalities and USD 3.00 billion worth of damage. Most of the fatalities were reported in France. Some fatalities also occurred in Germany, Spain, Portugal, and Belgium. Approximately one million homes in Western France experienced power outages. Falling trees caused damage of vehicles, houses, and other properties. Spain and France experienced the strongest winds of Xynthia, excessing 140 mph. Flooded railway tracks were reported in Spain, resulting in significant passenger and freight rail service disruptions. More than 70 flights were cancelled at the Paris–Charles de Gaulle Airport (France). The French government decided to assist French citizens and allocated certain monetary compensations for the property damage caused by Storm Xynthia.

#### 4.2.3. Storm David

Storm David formed on 16 January 2018 in the vicinity of Newfoundland (Canada). On 17 January, David approached Ireland and started intensifying afterward. Some force winds of up to 95 mph were recorded in Britain on 18 January. Then, the storm moved toward Western Europe, reaching a minimum central pressure of 974 hPa and a maximum wind speed of up to 126 mph [55]. The peak intensity was observed in Germany. David began weakening in the evening of 18 January when approaching the Polish borders. The storm fully dissipated on 22 January near Western Russia. As indicated earlier, Storm David caused 15 fatalities and USD 2.60 billion worth of damage. The following areas were affected by David: Ireland, United Kingdom, Germany, France, Switzerland, Italy, Poland, Ukraine, Russia, and others. The highest considerable damage was recorded in Germany, including 10 fatalities, approximately USD 1 billion worth of property damage and approximately 240,000 power outages. Trees were thrown around by the storm, blocking roadways and creating traffic congestion in some areas. Many airports and railway stations had to cancel their services due to the impact of David. Even in its weakened state, Storm David resulted in traffic disruptions, infrastructure damage, and more than 50,000 power outages.

### 4.3. Typhoons in Asia

In the context of Asian countries, China is often influenced by tropical cyclones. Recently, the average loss per year was reported to be over 10 billion yuan due to tropical cyclones. From a geographical perspective, Macau and China are advantageous to analyse typhoons due to monsoon seasons, shore-side regions, and subtropical climate. Macau and Hong Kong are located in the Pearl River Delta where both cities are close together (i.e., 38 miles of air travel distance) (https://www.distancefromto.net/, accessed on 1 March 2020). In this sense, Macau and Hong Kong were identified as illustrative tropical cyclones in Asia. Table 5 summarises the historical storms in Macau and Hong Kong since 1960. Number 8 signal (i.e., storm or gale), Number 9 signal (i.e., rising storm or gale), and Number 10 signal (i.e., typhoon) are recognised as strong tropical cyclones [16,64].

A total of 65 storms have been reported in Macau and Hong Kong since 1960. Interestingly, at least 1 strong tropical cyclone occurred in Macau or Hong Kong annually. Regarding the storm level, numbers 8, number 9, and number 10 contributed to 68%, 11%, and 21% of storms from 1960 to 2019, respectively. Studying the last six decades, a serious tropical cyclone period was reported from 1960 to 1969. In addition, the frequency of storms was reducing from 1970 to 2009. However, it began to increase again in 2010.

Table 6 shows that Hato, Mangkhut, and Vicente are the costliest storms that happened after 2000 in Hong Kong and Macau. The damage induced by these storms totalled around USD 10.53 billion. Storm Hato can be described as the deadliest one as it led to 12 deaths in Macau and the highest total monetary losses. Furthermore, Storm Nuri had the lowest wind speeds of up to 115 mph, while Storm Mangkhut had the highest wind speeds of up to 180 mph. In addition, the lowest pressure was recorded for Hato. Another important pattern consists in those two out of five costliest storms since 2000 that happened in 2017 and 2018 and caused over USD 10.1 billion worth of damage. Indeed, Hato caused considerable damage in Macau in 2017, and Mangkhut in Hong Kong was damaged severely in 2018. Low air pressure and high wind speeds were observed for the strongest tropical storms in Asia. In the following sections of the manuscript, we provide more information concerning some of the most severe storms in Macau and Hong Kong (in terms of fatalities and wind speed), including Hato, Mangkhut, and Vicente.

#### 4.3.1. Hato

Storm Hato has first discovered a nearshore approximately 75 km southeast of Zhuhai City on 23 August 2017. The highest wind speed close to the midpoint was 48 meters in an average of a second, the lowest pressure of the midpoint was 945.4 hPa, and the wind force reached a level exceeding 15. The area of the 10-level wind circle ranged between 70 and 80 km, and the area of the 12-level wind circle was 50 km. At 12:50 p.m., Storm Hato finally landed in Zhuhai City (i.e., the southern coast). As the wind force has reached the level of over 14 and the highest wind speed close to the midpoint was 45 meters in an average of a second, the typhoon hit the whole of Zhuhai City unexpectedly. Consequently, almost all trees were destroyed by the strong wind. Concrete poles and T-shaped steel rods were interrupted and demolished by the strong wind. Surprisingly, storm Hato has been recorded as the number 10 typhoon signal after the occurrence of typhoon York in 1999 [11,64].

#### 4.3.2. Mangkhut

Storm Mangkhut was initially found in Taishan City on 16 September 2018 at 6:00 a.m. The distance between Taishan City and the northeast sea of the South China Sea was around 420 km. The highest wind speed close to the midpoint was 50 meters in an average of a second, the lowest pressure of the midpoint was 956.4 hPa, and the wind force reached a level that was over 15. The area of the 10-level wind circle ranged between 150 and 270 km. Indeed, the area of the 12-level wind circle ranged from 60 to 80 km. It is unusual for the Central Meteorological Observatory to terminate its numbering due to the power diminishing, and the typhoon movement midpoint was difficult to recognise. The effect of rain and wind was severe enough to bring serious damage to numerous cities, including Macau, Hong Kong, and western, eastern, and southern Guangdong. In particular, outdoor facilities and buildings, such as bus stop poles, billboards, scaffolds, stilt houses, open sections of railways, farmlands, and fish ponds, were entirely demolished and severely knocked down [11,64].

#### 4.3.3. Vicente

Typhoon Vicente formed at 0:02 a.m. on 24 July 2012, approximately 90 km south-southeast of Macau. A series of strong winds were generally blowing in Macau and Hong Kong. The highest wind speed reached 140 mph. The minimum air pressure was 964.2 hPa. In general, Hong Kong and Macau suffered a significant impact from Typhoon Vicente. In Macau, the Civil Defense Center received at least 98 accident reports, 10 residents were injured, 5 flooding events were observed, 5 scaffolding structures collapsed, 3 scales crumbed, 23 trees were felled, and 10 aluminum accidents (i.e., the falling of windows and 10 advertising signs) were recorded. In Hong Kong, approximately 8800 trees were destroyed. Furthermore, 268 people sought storm shelters, and approximately 140 injured people sought medical treatment in Hong Kong [64].

## 5. Discussion

**Figure 5:**
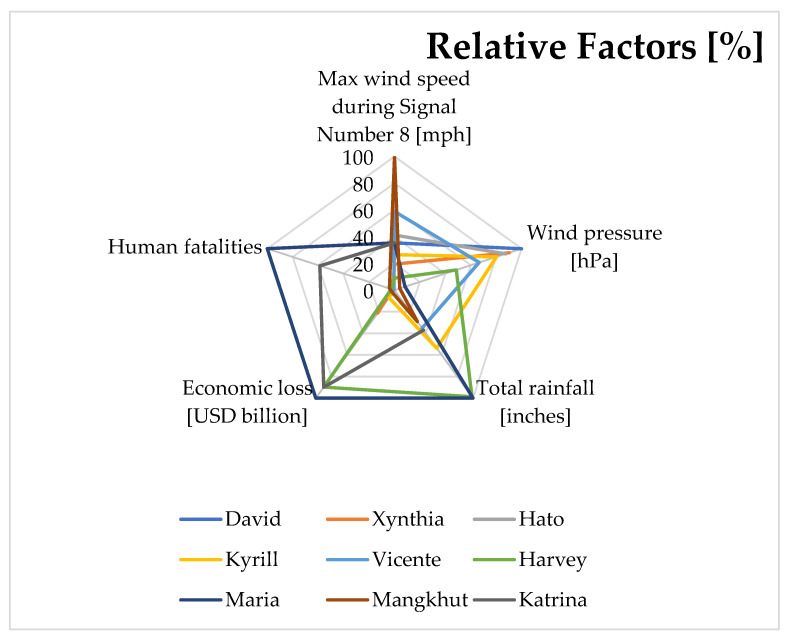
Summary of tropical cyclones in the USA, Europe, and Asia.

As a summary of this research, the study provides Figure 5 to highlight the nine tropical cyclone incidents in different aspects, including maximum wind speed, wind pressure, total rainfall, economic loss, and human fatalities. 

Based on a detailed review of the hurricanes in the USA, storms in Europe, and typhoons in Asia, the common features have been identified as follows: (1) large variations in terms of intensity; (2) strong follow-up rainfall; and (3) travel paths and landing points that are extremely favourable for strong impacts [65]. Yang et al. (2018) [5] pointed out that tropical cyclones could bring catastrophic risks to human welfare. Indeed, hurricanes, storm surges, and typhoons that occurred after 2000 have caused a significant number of fatalities and resulted in substantial property and facility damage (Figure 5). In this regard, Chen et al. (2021) [39] indicated an upward trend of intensification rates of strong tropical cyclones across the world. The rationale behind the rapid growth is increasing population, anthropogenic warming, and industrialisation. Coastal areas, low-lying coastlines, and inland areas may face a higher risk due to tropical cyclones [39,66]. The World Meteorological Organization [67] identifies the risk as ‘expected loss of lives, people injured, damage to property and disruption of economic activities due to a particular hazard in a given area and referenced period’. In other words, the risk can be defined as a chance of developing a dangerous action (or process) that will negatively affect the property, society, economy, environment, and people [68]. 

Table 7 summarises some of the main features of tropical cyclones in the USA, Europe, and Asia since 2000. Findings suggest that the USA and Europe have faced the highest frequencies of tropical cyclones, whereas Asia encountered the lowest frequencies of tropical cyclones. Munich RE (2020) [69] indicated that the USA suffered a large number of deaths and the highest level of losses due to the high frequencies of thunderstorms. In addition, according to several other studies, the conditions favourable for tropical cyclone formation in the USA and Europe may be related to the large sea surface with a higher temperature, the existence of the Coriolis force sufficient to produce a cyclonic vortex, the small changes in the vertical wind speed and the pre-existing weak low-pressure areas [70,71]. Interestingly, the 7 most severe storms occurred in the USA during the autumn season and the 8 most severe storms happened in Europe during the winter season. This scenario could explain that the storms could have destructive impacts due to wet snow, coastal flooding, and high winds. In general, severe storm events in the winter season will last longer than those in the summer season. The extended duration suggests extensive damage potential. With the effect of high-pressure systems, the winter season creates larger energetic waves along the coastal regions [72]. In addition, at the onset of autumn, the wind directions at both the high and low altitudes are relatively consistent, and the configuration is more favourable to the formation of typhoons with respect to the upper and lower layers’ atmospheric circulation in summer (i.e., the heating or cooling of the air at both high and low altitudes are relatively consistent with relatively small disturbance or interference). 

An increase in the water flow due to heavy rain during a strong tropical cyclone will lead to more and more flooding events. In total, 20–40% of Taiwan and South-eastern China’s annual rainfall is brought by tropical cyclones, and annual contributions of 10% have been reported over Florida. Over the Philippines, tropical cyclones contribute more than 50% over the northern regions and 6% in the southern region [73]. To this end, Fang et al. [74] pointed out that storm surges and precipitation are the two main forces of flooding. The World Health Organization [75] addressed that extreme rainfall may give rise to more floods. Flooding brings remarkable and extensive effects on health ranging from mental-health problems to injuries. Excessive precipitation incidents and a rising number of regional flooding incidents have intensified the risk and addressed the spread of numerous infectious diseases which could potentially influence their chances and distribution of becoming epidemics. Moreover, excessive rainfall will create tanks of stagnant water that generate growth environments for hosts and vectors such as insects, rodents, mites, and mosquitoes [76]. Due to heavy rainfalls, the flooding of health facilities would seriously damage infrastructures of electrical power and water supply, and increase the difficulty in offering normal care for patients with chronic diseases [75]. Indeed, low-lying areas covered with muddy waters comprise a large quantity of silt and clay from soil erosion after heavy rainfall [77]. Heavy rainfalls will put up barriers to providing emergency logistics in the low-lying areas. Nevertheless, Kim et al. [78] criticised the idea that the interface of the typhoon with the land and the regional terrain reduces accurate rainfall forecasting. As a result, it will decrease emergency response and disaster preparedness. 

In addition, high-intensity storms quickly damage major roads and lead to traffic congestion. As a result of traffic congestion, many vehicles on the roads experience higher levels of noise and air pollution on average. Moreover, high-intensity storms will adversely influence the construction of green transportation infrastructure, reduce transportation efficiency, decrease accessibility to the transportation networks, and increase transportation costs. Besides, the coastal areas are vulnerable to natural disasters, such as hurricane-induced flash floods. Highly urbanised and densely populated areas with infrastructure along coastal areas are more reactive to natural disasters [79]. Indeed, higher sea levels reduce the drainage rate of the coastal areas, which further causes more flooding due to hurricanes and typhoons. Along with substantial damage to the infrastructure, strong tropical cyclones destroy beaches and wetlands.

In this study, we have investigated the consequences of ocean floods, flash floods, and storm surges due to tropical cyclone activities in the USA, Europe, and Asia. Moreover, the frequency of tropical cyclone incidents in coastal regions seems to be rising in the considered regions. Furthermore, the infrastructure is showing a tendency to be more vulnerable to climate and weather extremes, which are intensified even further by climate change [1]. The degree of damage from tropical cyclones often varies depending on the infrastructure type, geographical location, and port or shipping business, which means that some regions can be more susceptible to the devastating impacts of hurricanes and typhoons as compared with other regions. As such, we need to take steps to increase the awareness of climate change impacts on tropical cyclone incidents, coupled with designing resilience and adaptation strategies in response to tropical cyclones in the coming years. Different strategies for slowing down climate change should be explored (e.g., emission reduction strategies [80,81,82]). On the other hand, the appropriate planning of humanitarian logistics can help lower the risks and reduce the tropical cyclone severity. In particular, the industrial practitioners, local communities, researchers, and policymakers [83] may need to reinforce the idea of humanitarian logistics during the introduction and growth stage of tropical cyclones. Early warning via advanced communication technology and hazard monitoring for risk assessment would be able to minimise the risk, human fatalities, and economic losses in the future. Bouwer and Jonkamn [84] and EM-DAT [85] also indicated that global mortality has been declining over time because of enhancements in humanitarian logistics and disaster risk management. Humanitarian logistics deals with the cyclones at different phases of the cyclone’s events, such as the post-cyclone phase. The knowledge of humanitarian logistics can better minimise the response time, manage the delivery of goods and organise rescue activities.

## 6. Conclusions

In this study, we have carried out tropical cyclone research and reviewed the major tropical cyclone activities in the USA, Europe, and Asia. In this manner, we can make a comparative analysis of tropical cyclones across geographical regions. This approach may provide useful guidelines and adaptation measures in response to the different levels of storms. Through examining the meteorological contexts and the major attributes of typhoons and hurricanes (i.e., the year of occurrence, maximum wind speed, lowest pressure, total deaths, total monetary losses, and total rainfall), we determined the main meteorological factors that caused disasters and their consequences. 

The conducted review revealed that Katrina, Harvey, and Maria were the costliest hurricanes in the USA since 2000, which caused almost USD 380 billion of property damage between them. Kyrill, Xynthia, and David were found to be the costliest storms in Europe with the total property damage exceeding USD 10.3 billion. Moreover, Hato, Mangkhut, and Vicente were identified as the costliest storms in Hong Kong and Macau [86], which resulted in USD 10.53 billion of property damage. Therefore, the costliest and deadliest storms were found to be more common in the USA rather than in Europe and Asia. Hurricane Maria was found to be the deadliest tropical cyclone that caused over 3000 fatalities in 2017. This study revealed that many devastating tropical cyclones have occurred in the last 3 to 4 years, which highlights the urgency of the issue of climate change. The wind speeds and heavy precipitation are expected to increase, accompanied by an increase in the frequency of severe storms. According to EM-DAT (2022) [85], the impacts of cyclones on storm surge and the amount of precipitation are critical in some regions, such as inland regions and low-lying riverbank areas. Moreover, findings showed that the total deaths and total losses were proportioned to the frequency and power of tropical cyclone incidents. The rapid growth of industrialisation, urbanisation, and various human activities (e.g., building and businesses) accelerate climate change. As such, Lee et al. (2020) [87] has already reported the trend analysis by the Typhoon Committee. The study identified an inclination to change from tropical depressions (i.e., weak tropical cyclones) into hurricanes and typhoons due to the effect of rapid climate change in Asia, Europe, and the USA

Effective measures have to be undertaken by all sectors to prevent drastic climate changes that cause the formation of catastrophic hurricanes and typhoons, such as the use of renewable energy [88,89]. Furthermore, the present research highlighted the urgency for the design and implementation of effective resilience strategies against potential impacts that could be caused by major tropical cyclones (e.g., severe storms, heavy rainfall, and floods). A substantial portion of the public sector still does not realise the importance of resilience strategies, as they have insufficient awareness of potential threats that could result from tropical cyclones. Innovative technologies and educational programs should be implemented to facilitate public awareness regarding climate change and the potential consequences of hurricanes and typhoons. Human life threats and their health risks can be minimised in the future [86].

As for future research extensions, another research can be dedicated to a future trend analysis of tropical and extra-tropical cyclones for each region. In this manner, the anticipated future impacts can be compared, and new conclusions can be drawn. New prediction models may be developed to quantify the climate change impacts on the considered geographical regions. Moreover, future research may investigate additional factors that can potentially influence the post-landfall devastation (e.g., landfall location, urban/rural designation of the affected area, and population of the affected area). In addition, a comparative analysis can be conducted to assess the socioeconomic differences (e.g., average income per household, the perceived value of infrastructure, and unemployment percentage) across various regions experiencing tropical cyclones (i.e., Asia, Europe, and USA). Based on the collected data, applied econometric models can then be developed. Such an analysis is expected to provide more insights into the major tropical cyclones across different regions. Furthermore, we will record direct and indirect deaths via focus group interview(s) to investigate how people perceive tropical cyclones in response to the direct and indirect impact, as well as direct and indirect deaths, resulting from tropical cyclones. 

## Figures and Tables

**Table 1:** Typhoon data as logistics attributes.

Item	Logistics Attributes	Typhoon Data
1	Location	Formation location Landing location (destination) Maturation location (e.g., typhoon Number 8) Decline location (e.g., lowered the signal)
2	Transport	Date of year Season of year Time of formulation Moving speed Routing Duration of Number 8
3	Inventory	Wind speed or Wind scale Air pressure Rainfall Temperature (i.e., versus average temperature of year) The radius of the wind circle
4	Value	Economic loss Deaths and injuries

**Table 2:** The interface between a typhoon event and humanitarian logistics.

Stage	PLC	Humanitarian Logistics
1	Introduction (or formation)	Early warning through advanced communication technology
2	Growth (or development)	Hazard monitoring for risk assessment
3	Maturity	Prevention; business continuity plan (e.g., mobilising relief items, such as water, food, and housing); quick transportation; allocation of scarce resources
4	Decline	Disruptions and recovery via post-disaster humanitarian operations

**Table 3:** Costliest hurricanes in the USA since 2000.

a/a	Hurricane	Year	Maximum Wind Speed (mph)	Lowest Pressure (hPa)	Total Deaths	Total Losses (USD)	Total Rainfall (inches)
1	Katrina	2005	175	902	1836	160.0 billion	37
2	Harvey	2017	130	937	107	125.0 billion	99
3	Maria	2017	175	908	3059	91.6 billion	100
4	Irma	2017	180	914	134	77.2 billion	15
5	Sandy	2012	115	940	233	70.2 billion	10
6	Ike	2008	145	935	214	38.0 billion	10
7	Ivan	2004	165	910	124	27.1 billion	7
8	Michael	2018	160	919	74	25.1 billion	7
9	Wilma	2005	185	882	52	24.3 billion	6
10	Florence	2018	150	937	54	24.2 billion	10

**Table 4:** The list of costliest storms in Europe since 2000.

a/a	Storm	Year	Maximum Wind Speed (mph)	Lowest Pressure (hPa)	Total Deaths	Total Losses (USD)	Total Rainfall (inches)
1	Kyrill	2007	160	960	53	4.70 billion	54
2	Xynthia	2010	142	967	63	3.00 billion	16
3	David	2018	126	974	15	2.60 billion	21
4	Ciara	2020	136	943	13	1.90 billion	14
5	Gudrun	2005	103	960	12	1.38 billion	9
6	St. Jude	2013	121	967	17	1.10 billion	10
7	Xaver	2013	142	962	15	1.00 billion	10
8	Eleanor	2018	140	966	6	0.64 billion	5
9	Lorenzo	2019	160	925	19	0.36 billion	6
10	Andrea	2012	109	964	1	0.35 billion	8

**Table 5:** Recorded storms that occurred in Macau and Hong Kong (1960–2019).

	1960–1969	1970–1979	1980–1989	1990–1999	2000–2009	2010–2019
**Number 8 signal**	8	6	8	8	7	7
**Number 9 signal**	1	2	0	2	0	7
**Number 10 signal**	6	3	1	1	0	3
**Total**	15	11	9	11	9	10

**Table 6:** Summary of the most severe storms in Macau and Hong Kong since 2000.

a/a	Storm	Year	Maximum Wind Speed (mph)	Lowest Pressure (hPa)	Total Deaths	Total Losses (USD)	Total Rainfall (inches)
1	Hato	2017	132	945.4	12 (Macau)	USD 6.41 billion	50
2	Mangkhut	2018	180	956.4	0	USD 3.77 billion	29
3	Vicente	2012	140	964.2	0	USD 0.35 billion	35
4	Dujuan	2003	145	950	0	USD 0.31 billion	67
5	Nuri	2008	115	955	2 (Hong Kong)	USD 0.085 billion	52

**Table 7:** Summary of tropical cyclones in the USA, Europe, and Asia since 2000.

Region	Total Number of Storms	Season	Levels of Tropical Cyclones	Total Deaths	Total Losses (USD)	Total Rainfall (inches)
USA	10	3 Summers 7 Autumn	10 super typhoons	5887	USD 662.7 billion	301
Europe	10	2 Autumns 8 Winters	5 typhoons 3 severe typhoons 2 super typhoons	214	USD 17.03 billion	153
Asia	5	4 Summers 1 Autumn	1 typhoon 3 severe typhoons 1 super typhoon	14	USD 10.93 billion	230
